# Minimal impact of extensive heating of hen's egg and cow's milk in a food matrix on threshold dose‐distribution curves

**DOI:** 10.1111/all.13198

**Published:** 2017-05-26

**Authors:** B. C. Remington, J. Westerhout, D. E. Campbell, P. J. Turner

**Affiliations:** ^1^ TNO Zeist The Netherlands; ^2^ Department of Allergy & Immunology Children's Hospital at Westmead Sydney Australia; ^3^ Discipline of Child and Adolescent Health University of Sydney Sydney Australia; ^4^ Section of Paediatrics (Allergy and Infectious Diseases) & MRC and Asthma UK Centre in Allergic Mechanisms of Asthma Imperial College London London UK

**Keywords:** Cow's milk, food allergy, food challenge, hen's egg, minimal eliciting dose

## Abstract

We analyzed reaction threshold data from 352 children undergoing open food challenges to hen's egg or cow's milk, either fresh or extensively heated into a muffin. There was no significant shift in dose‐distribution curves due to the baking process, implying that existing threshold data for these allergens can be applied to allergen risk management, even when these allergens are heat‐processed into baked foods.

## 1 INTRODUCTION

Up to 70% of children with IgE‐mediated allergy to egg and cow's milk (CM) are able to tolerate the allergen when extensively heated into a baked food (such as cakes and biscuits).^1^, ^2^, ^3^, ^4^, ^5^ In addition to liberalizing the diet (and potentially reducing anxiety over accidental ingestion), ongoing consumption of the allergen in baked foods may alter the natural history of the disease and accelerate the acquisition of tolerance.^5^ It has been postulated that tolerance to “baked” egg and CM is related to conformational changes induced by heating,^5^ but could also be due to the amount of egg/CM protein found in most baked products being below the amount (“threshold”) needed to trigger symptoms in tolerant individuals.

An expert panel recently proposed that dose‐distribution data relating to reaction thresholds should be used to inform the advisory cutoffs for “may contain” precautionary advisory labeling (PAL).^6^ However, existing data for egg and CM are from oral food challenges (OFC) performed with fresh egg and CM. The effect of food processing (such as baking) has not been reported, and this information is important. CM was detected in 43% of German bakery products labeled as “milk‐free,” with 21% of foods tested estimated to contain sufficient allergen to provoke an allergic reaction in at least 10% of milk‐allergic children based upon known thresholds for nonextensively heated allergen.^7^

While data exist that average eliciting doses for reactions to baked egg and CM at OFC are higher than those to the native allergen,^1^, ^3^, ^4^ these data are limited by cohort size and subject to selection bias (where many included children did not undergo OFC to the native allergen). Reactions to baked egg or CM are often more severe than those reported to the native allergen at food challenge, with anaphylaxis a relatively common outcome.^1^, ^2^, ^8^ Indeed, up to one in three reactions to baked CM at OFC are associated with anaphylaxis,^4^ and fatal reactions to baked CM have been reported (personal communication). Allergic individuals unable to tolerate egg and CM in baked foods may represent a more severe phenotype 4 and could react to lower levels of exposure compared to those who tolerate the baked allergen.

It is therefore important to determine whether eliciting doses for egg and CM in baked food are comparable to those for the native allergen. In this study, we examined allergen threshold data from OFC undertaken in children allergic to baked egg or CM, and compared these to similar data derived from positive OFC to the native allergen undertaken during the same time period, within the same clinic population.

## 2 METHODS

The Children's Hospital at Westmead is a major tertiary pediatric allergy center in Australia, undertaking approximately 1000 OFC annually. Children presenting consecutively to our clinic between 2009 and 2016 with a clinical diagnosis of allergy to CM or egg, and who were following complete dietary elimination of these allergens, were offered an OFC to the extensively heated allergen in a muffin. Open OFC were performed as previously described using a standardized recipe for baked egg‐ and CM‐containing muffins, standard dosing regime, and stopping criteria,^1^, ^2^ consistent with the PRACTALL consensus to determine objective symptoms.^9^ For whole egg OFC, the egg underwent minimal heating in a microwave (600 W, 2×30 seconds periods) to create “lightly scrambled” egg. In 2015, a lower starting dose of protein (1 mg) was added to the OFC protocols to reduce the number of first‐dose reactions. The study was approved by the local ethics committee, and written consent was obtained for all OFC.

Individual no observed adverse effect level (NOAELs) and lowest observed adverse effect level (LOAELs) were determined from the cumulative dose causing an objective reaction, and interval‐censoring survival analysis (ICSA) was utilized to generate statistical dose‐distribution curves (log‐normal, log‐logistic, Weibull) as previously described.^10^ The ED_10_ and ED_50_, or the eliciting doses respectively predicted to provoke reactions in 10% and 50% of the population, were estimated. For all analyses, the R software suite (https://www.r-project.org/) and Survival package (https://cran.r-project.org/web/packages/survival/index.html) were used.

## 3 RESULTS

**Figure 1:**
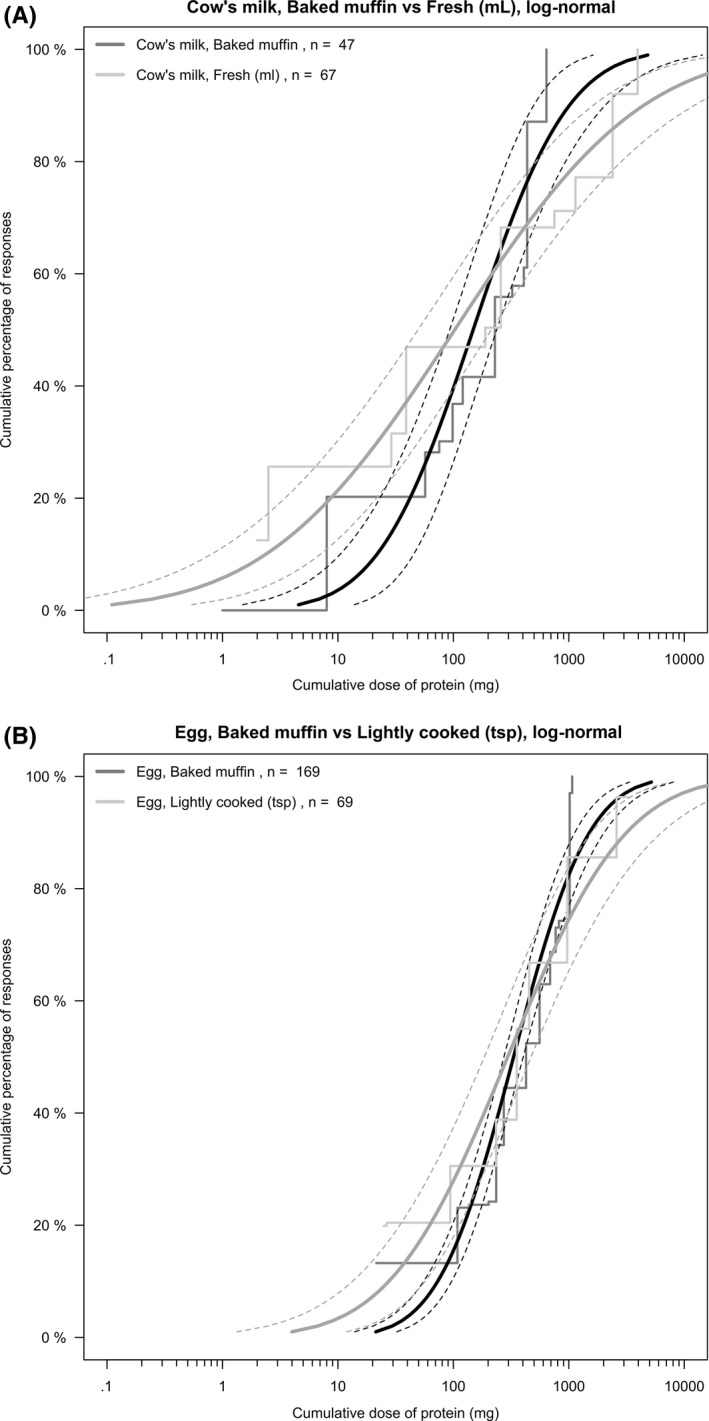
Log‐normal threshold distribution curves of (A) cow's milk‐ and (B) egg‐allergic children reacting with objective symptoms during oral food challenge to baked or “native” egg or cow's milk

Data were available for a total of 352 positive OFC to lightly cooked egg, CM, or a baked muffin containing either egg or CM (Table 1). The ED_50_, or the mg protein predicted to elicit objective symptoms in 50% of the population, predicted by the dose‐distribution curves for thresholds of reactivity to egg and CM were not altered significantly when the allergen was in baked form (Table 1). All statistical distributions (log‐normal, log‐logistic, Weibull) resulted in similar threshold distribution curves and estimations of the ED_50_, and the log‐normal threshold distribution curves are presented in Figure 1. The estimated ED_10_ for egg and CM in nonbaked foods were 19‐29 (95% CI: 7‐64) mg protein and 1.6‐2.7 (95% CI: 0.4‐8.8) mg protein, respectively.

**Table 1:** Combined ED50 range estimated by the log‐normal, log‐logistic, and Weibull distributions for oral food challenges using egg and cow's milk (CM; both in native form and “baked” into a muffin)

Allergen	Form	Number of individuals (left censored, right censored)	Predicted ED50 range
Egg	Lightly cooked	69 (15, 11)	296‐360 mg protein (95% CI: 185‐570 mg protein)
	Baked	169 (23, 37)	332‐384 mg protein (95% CI: 274‐453 mg protein)
CM	Fresh	67 (10, 3)	103‐157 mg protein (95% CI: 49‐319 mg protein)
	Baked	47 (9, 10)	148‐177 mg protein (95% CI: 93‐271 mg protein)

## 4 DISCUSSION

We did not find any significant impact of baking on dose‐distribution curves for egg or CM protein in this population of allergic children. This implies that firstly, existing dose‐distribution data can be applied when these allergens are present in baked food such as cake, and secondly, tolerance to baked egg or CM is unlikely to be due to the lower amounts of these allergens typically present in baked foods. There are some caveats to these data. Ideally, all children reacting to the baked allergen would have undergone a subsequent OFC to fresh CM and lightly cooked egg to directly compare extensively heated and “native” allergen within the same individuals. Goldberg et al.^11^ reported 13 children allergic to both fresh and baked CM, undergoing desensitization using baked milk; eight of 13 had a threshold to baked CM more than double that to fresh CM. However, outside the context of a desensitization study, we felt it unlikely that parents would agree to OFC where their child had reacted to a baked challenge to the same allergen, a reservation also raised by the other research groups^3^, ^4^ and by our ethics committee. We therefore sought to avoid this issue by comparing a challenge data from a large cohort of children undergoing baked allergen‐OFC to children from the same clinic population undergoing contemporaneous OFC to the “native” allergen. Importantly, all eligible children were offered an OFC to the baked allergen, to minimize possible selection bias.

These data were generated from routine diagnostic OFC, and as such, results are limited by the relatively high starting doses used for a number of these open challenges, particularly for baked allergen (approximately 60 mg protein). This resulted in a degree of “left‐censored” data (where individuals reacted to the first dose), which has been shown to significantly affect the lower ED estimates.^12^ We attempted to mitigate this effect by introducing a lower starting dose into the challenge protocol in 2015. The ED_10_ estimates obtained for lightly cooked egg and fresh CM are consistent with published data.^6^, ^13^, ^14^ Nonetheless, the data obtained in this analysis are generally above the ED_20_ values, and thus, apparent differences in the threshold distributions below the ED_20_ should not be given too much significance. More data at lower doses would help confirm the similarity or elucidate a difference between the allergen in “native” and “baked” form.

We found that the dose‐distribution curves for reaction thresholds to lightly cooked egg and fresh CM were not significantly different to those obtained from OFC using extensively heated allergen. This implies that individuals who are allergic to extensively heated allergens are unlikely to have a lower triggering dose (ie, are more “sensitive”) compared to allergic subjects who tolerate the allergen in baked foods. On the basis of these data, it would seem a reasonable approach to use existing threshold data for egg and CM in allergen risk management, including with respect to the need for PAL, when these allergens are processed into baked foods—however, the collection of further data on individuals who react at very low doses of baked allergen is desirable.

## CONFLICTS OF INTEREST

Benjamin C. Remington and Joost Westerhout are employed by TNO, the Netherlands. TNO is a nonprofit R&D organization which collaborates with both public and private stakeholders for research or consultations related to food allergy. The analyses presented in this work were not influenced by these various collaborations. All authors otherwise declare no conflict of interests.

## AUTHOR CONTRIBUTIONS

DEC and PJT developed the concept and proceeded with data collection. All authors contributed to the analysis presented, contributed to drafting the article, and approved the final version.
